# Identification of novel germline and somatic mutations associated with hepatocellular carcinoma by next-generation sequencing

**DOI:** 10.3389/fphar.2025.1699280

**Published:** 2026-01-06

**Authors:** Ahmed Baligh Laaribi, Wafa Babay, Abdelmalek Lekired, Bochra Bouchabou, Asma Mehri, Dhouha Bacha, Riadh Sassi, Sana Ben Slama, Rached Bayar, Sahir Omrani, Nadia Boujelbene, Rim Ennaifer, Nafaa Arfa, Ahlem Lahmar, Hadda-Imene Ouzari

**Affiliations:** 1 Laboratory of Microorganisms and Active Biomolecules (LR03ES03), Faculty of Sciences of Tunis, University of Tunis El Manar, Tunis, Tunisia; 2 Common Sequencing Unite, Faculty of Sciences of Tunis, Department of Biology, University of Tunis El Manar, Tunis, Tunisia; 3 Department of Gastroenterology, University Hospital of Mongi Slim, La Marsa, Tunisia; 4 Department of Pathology Anatomy, University Hospital of Mongi Slim, La Marsa, Tunisia; 5 Department of General Surgery and Liver Transplantation, University Hospital of Mongi Slim, La Marsa, Tunisia

**Keywords:** cancer genomic, germline mutations, hepatocellular carcinoma, next-generation sequencing, precision medicine, signalling pathways, somatic mutations

## Abstract

**Background:**

Hepatocellular carcinoma (HCC) is the third leading cause of cancer-associated deaths worldwide with an estimated of 900,000 new cases annually. HCC typically arises in patients with chronic liver disease, including hepatitis, cirrhosis, and non-alcoholic fatty liver diseases. Identifying of the main driver genetic alterations in oncogenic genes is essential for understanding HCC pathogenesis and defining prognostic biomarkers in high-risk patients. This study aimed to identify both germline and somatic mutations associated with HCC in a Tunisian patient’s cohort.

**Methods:**

Forty HCC patients with different etiologies were included in this study. Peripheral blood samples were collected from 24 patients with advanced-stage HCC. Paired tumor and adjacent non-tumoral liver tissue samples were obtained from 16 early-stage HCC patients undergoing hepatic resection, including 10 fresh-frozen samples and 6 FFPE samples. DNA was extracted using the MagCore® Plus II system. Targeted next-generation sequencing was performed using *the Illumina AmpliSeq™ Cancer Hotspot Panel v2*.

**Results:**

A total of 35 germline mutations were identified across 25 genes. Recurrently altered genes included FGFR3 (100%), PDGFRA (100%), RET (98%), APC (92%), TP53 (88%), and EGFR (75%). In addition, 14 somatic mutations were detected in 13 genes, with frequent alterations observed in APC (100%), ALK (94%), HNF1A (56%), CDKN2A (50%), and HRAS (50%).

**Conclusion:**

This study offers the first comprehensive overview of novel germline and somatic mutations in Tunisian HCC patients, representing a North African cohort, and highlights key molecular drivers of hepatocarcinogenesis. These findings support the integration of genetic profiling into clinical practice to enhance early diagnosis and guide personalized therapies.

## 1 Introduction

Hepatocellular carcinoma (HCC) is the most common form of primary liver tumour and is ranks as the third cause of cancer-related deaths worldwide (Rumgay et al., 2022). The major risk factors for HCC development are; chronic hepatitis B or C infections, nonalcoholic fatty liver disease (NAFLD), diabetes mellitus, chronic alcoholic consumption, aflatoxin B1 (AFB1), aristolochic acid (AA) and others causes of cirrhosis such as genetic or metabolic liver diseases (Yang et al., 2019). HCC present a major global health burden, with more than 800,000 new cases are diagnosed annually and over 750,000 deaths reported each year. The disease is characterized by a poor prognosis, particularly in patients diagnosed at advanced stages (Bray et al., 2024). Current treatment strategies, ranging from curative approaches such as surgical resection and liver transplantation to palliative modalities including loco-regional and systemic therapies, remain suboptimal for many patients. This highlights the urgent need for improved diagnostic tools and the development of more effective therapeutic strategies to enhance clinical outcomes and survival (Singal et al., 2023).

Understanding the molecular mechanisms underlying HCC is essential for the development of novel biomarkers and targeted molecular therapies. Over the past decade, next-generation sequencing (NGS) technologies have significantly advanced our understanding of cancer genomics and have revolutionized the molecular characterization of HCC. These high-throughput approaches allow comprehensive profiling of somatic and germline mutations, copy number alterations, gene expression changes, and epigenetic modifications (Chung et al., 2023). Large-scale sequencing efforts have identified recurrent alterations in key oncogenes and tumor suppressor genes, such as *TP53*, *CTNNB1*, *AXIN1*, and *TERT* promoter mutations, as well as dysregulation of critical signalling pathways including Wnt/β-catenin, PI3K/AKT/mTOR, and MAPK (Müller et al., 2020). Despite these discoveries, few biomarkers have been clinically validated for HCC, which limits the widespread application of precision oncology in clinical practice. This limitation underscores the need to further investigate the landscape of accumulating genetic aberrations in liver cancer cells (Bruix et al., 2015; Llovet et al., 2024).

Furthermore, a significant gap remains in the comprehensive molecular characterization of HCC in underrepresented populations. Most available genomic data are derived from Asian or Western cohorts, while the genetic landscape in populations such as North Africans remains largely unexplored (Rotimi et al., 2021). This lack of representation may compromise the generalizability of molecular biomarkers and impede the development of personalized therapeutic strategies.

In this context, the present study aims to investigate the mutational spectrum of both somatic and germline alterations in Tunisian patients with HCC with different etiologies using targeted NGS technology. By employing an optimized panel of clinically relevant cancer-associated genes, our objective is to identify population-specific variants, reveal potential therapeutic targets, and contribute to a more comprehensive and inclusive understanding of HCC molecular pathogenesis.

## 2 Materials and methods

### 2.1 Patients and clinical samples

This retrospective study included 40 Tunisian patients diagnosed with HCC. It was conducted in accordance with the ethical principles of the Declaration of Helsinki and approved by the Ethics Committee of the University Hospital of Mongi Slim (approval number: P2ES 31/2021). The study period spanned from December 2021 to January 2023. Written informed consent was obtained from all participants prior to inclusion.

Ten surgically resected HCC tissue samples, along with their matched adjacent non-tumor liver tissues, were collected from patients who underwent curative hepatic resection. Tissue sampling was performed with the assistance of a certified pathologist. Immediately after resection, the samples were preserved in RNAprotect® Tissue Reagent (QIAGEN, Hilden, Germany) and stored at −20 °C until nucleic acid extraction.

In addition, six HCC samples were obtained as formalin-fixed, paraffin-embedded (FFPE) tissues, and histopathological confirmation of HCC was performed according to standard diagnostic criteria.

Furthermore, peripheral blood mononuclear cells (PBMC) samples were collected from 24 HCC patients who were not eligible for surgical treatment and were instead referred to the Department of Gastroenterology for palliative oncologic therapy or best supportive care. Whole blood was collected in EDTA tubes and stored at −80 °C until further molecular analysis.

### 2.2 Genomic DNA extraction and quality control

Genomic DNA was extracted using the MagCore® Plus II automated extraction system (RBC Bioscience Corp., New Taipei City 23145, Taiwan). For whole blood samples, the MagCore® Genomic DNA Whole Blood Kit (Cartridge Code: 102) was used, while the MagCore® Genomic DNA Tissue Kit (Cartridge Code: 401) was employed for DNA extraction from both fresh-frozen and FFPE tissue samples. All extractions were performed according to the manufacturer’s instructions.

DNA concentrations were quantified using the Denovix QFX Fluorometer (DeNovix Inc., Wilmington, DE, United States) in combination with the Qubit dsDNA High Sensitivity Assay Kit (Thermo Fisher Scientific, Waltham, United States). DNA quality and integrity were assessed using the Agilent 2100 Bioanalyzer (Agilent Technologies, Santa Clara, CA, United States).

### 2.3 Targeted next-generation sequencing

For targeted sequencing, genomic DNA (gDNA) libraries were constructed using 100 ng of input DNA per sample. Library preparation was performed with the AmpliSeq™ for Illumina® Cancer Hotspot Panel v2 (Illumina Inc., San Diego, CA, United States), which targets 2,800 known hotspot mutations across 207 amplicons in 50 cancer-related oncogenes and tumor suppressor genes, as listed in the Catalogue of Somatic Mutations in Cancer (COSMIC) database. These include: *ABL1, AKT1, ALK, APC, ATM, BRAF, CDH1, CDKN2A, CSF1R, CTNNB1, EGFR, ERBB2, ERBB4, EZH2, FBXW7, FGFR1, FGFR2, FGFR3, FLT3, GNA11, GNAS, GNAQ, HNF1A, HRAS, IDH1, IDH2, JAK2, JAK3, KDR, KIT, KRAS, MET, MLH1, MPL, NOTCH1, NPM1, NRAS, PDGFRA, PIK3CA, PTEN, PTPN11, RB1, RET, SMAD4, SMARCB1, SMO, SRC, STK11, TP53*, and *VHL*.

Briefly, amplicon libraries were prepared using multiplex PCR, followed by a digestion step to remove primer sequences. Adapter ligation was performed using the AmpliSeq for Illumina® CD Indexes Set A. Libraries were purified using Agencourt® AMPure® XP beads (Beckman Coulter Inc., Brea, CA, United States).

Library quantification was conducted using the DeNovix QFX Fluorometer, and amplicon size distribution was verified by agarose gel electrophoresis. Final libraries were sequenced on the Illumina® MiSeq platform using a 2 × 150 bp paired-end run. All procedures were carried out in accordance with the manufacturer’s protocols. Resulting sequence data were analysed using the BaseSpace™ Variant Interpreter (Illumina Inc.). All identified variants were manually inspected and confirmed by visualization within the Integrative Genomics Viewer (IGV).

### 2.4 Protein-protein interaction network construction and clustering analysis

To explore potential functional relationships among the proteins encoded by genes carrying germline and somatic mutations detected in our HCC cohort, the protein–protein interactions (PPI) network was constructed using STRING database (version 12.0; https://string-db.org/). Gene symbols were input into the STRING web interface, and the (PPI) network was generated with default parameters. For cluster detection within the PPI network, we applied the Markov Cluster Algorithm (MCL) with an inflation parameter set to the default value of 3. Cluster annotation was performed using the built-in functional enrichment tools in STRING, based on KEGG pathway annotations.

### 2.5 Statistical analysis

Statistical analyses were performed using GraphPad Prism version 9.0 (GraphPad Software, La Jolla, CA, United States). The landscape of genomic alterations was visualized using R (version 4.3.1) within RStudio (version 2024.12.0 + 467), employing the oncoPrint function from the ComplexHeatmap package.

## 3 Results

### 3.1 Demographic and clinical characteristics of study population

The demographic and clinico-pathological characteristics of the 40 HCC patients enrolled in this study (13 HBV-related, 13 HCV-related and 14 non-B/non- C (NBNC)) are summarized in Table 1. The cohort comprised 21 males (52%) and 19 females (48%), with a mean age at diagnosis ≥50 years in 87% of cases. Cirrhosis was present in 90% of patients.

**TABLE 1:** Demographic and clinicopathological characteristics of HCC patients.

Characteristics	HBV history N = 13	HCV history N = 13	Metabolic history N = 14	Total cases
Gender				
Male	8	6	7	21 (52%)
Female	5	7	7	19 (48%)
Age at diagnosis				
<50	3	1	1	5 (13%)
≥50	10	12	13	35 (87%)
Cirrhosis				
No	2	*1*	*3*	4 (10%)
Yes	11	*12*	*11*	36 (90%)
Tumor number				
1 nodule	5	7	3	15 (37.5%)
2 nodules	5	3	2	10 (25%)
≥3 nodules	3	3	9	15 (37.5%)
Tumor size (cm)				
< 2	8	5	4	17 (42.5%)
2–5	3	4	6	13 (32.5%)
≥ 5	2	4	4	10 (25%)
ALT				
≤40 U/L	5	4	4	13 (32.5%)
>40 U/L	8	9	10	27 (67.5%)
AST				
≤40 U/L	6	3	5	14 (35%)
>40 U/L	7	10	9	26 (65%)
AFP				
≤300 ng/mL	7	4	7	18 (45%)
>300 ng/mL	6	9	7	22 (55%)
BCLC				
A	3	4	2	9 (22.5%)
B	5	4	3	12 (30%)
C	2	1	4	7 (17.5%)
D	3	4	5	12 (30%)
Child pugh score				
A	7	5	3	15 (37.5%)
B	4	5	8	17 (42.5%)
C	2	3	3	8 (20%)

Regarding tumor characteristics, 37.5% of patients presented with a single nodule, while 25% had two nodules and 37.5% had three or more. Tumor size was <2 cm in 42.5% of cases, 2–5 cm in 32.5%, and ≥5 cm in 25%. Liver function tests showed elevated ALT (>40 U/L) and AST (>40 U/L) levels in 67.5% and 65% of patients, respectively. Serum alpha-fetoprotein (AFP) levels exceeded 300 ng/mL in 55% of the cohort.

According to the BCLC staging system, 22.5% of patients were classified as stage A, 30% as stage B, 17.5% as stage C, and 30% as stage D. Child-Pugh classification showed that 37.5% of patients were in class A, 42.5% in class B, and 20% in class C.

### 3.2 Germline mutations identified in HCC patients

**FIGURE 1:**
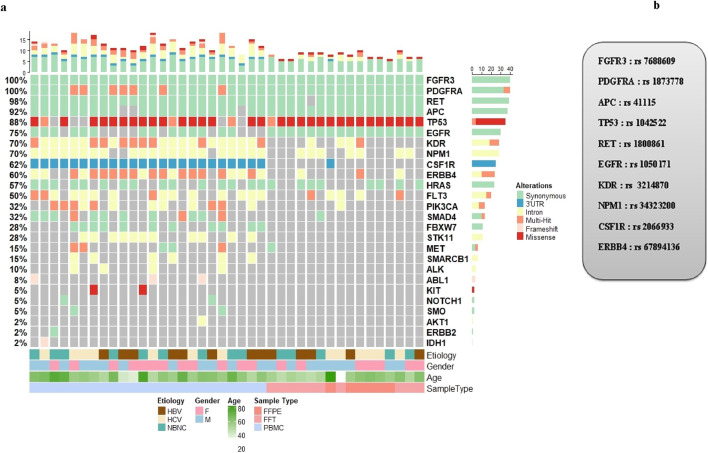
**(a)** Oncoplot displaying Hotspot oncogenic germline variants identified across HCC patients. Each column represents an individual, and each row corresponds to a cancer-associated gene. Only high-confidence germline variants that passed quality control filters are shown. The upper histogram shows the number of variants per patient, and the side histogram indicates gene-wise mutation frequency. Annotations below the plot represent clinical metadata including gender, age, sample type, and etiology (HBV, HCV, or non-viral). **(b)** List of the most frequently detected oncogenic hotspot germline mutations in our cohort of HCC patients.

A total of 40 samples from patients with HCC, including 24 PBMC samples and 16 paired HCC tumor and non-tumoral adjacent tissue (NTAT) samples, were analysed by NGS using the Ampliseq for Illumina Hotspot Panel v2 to identify germline mutations associated with HCC development in our cohort. Targeted screening of 50 oncogenic genes revealed 25 mutated genes, demonstrating a broad spectrum of genetic alterations, as shown in Figure 1a.

The most frequently mutated genes in all samples were FGFR3, PDGFRA and RET with mutation rates of 100%, 100% and 98%, respectively. Other highly mutated genes included APC (92%), TP53 (88%) and EGFR (75%), followed by KDR (70%), NPM1 (70%), CSFR1 (62%), ERBB4 (60%), HRAS (57%) and FLT3 (50%). Genes such as PIK3CA (32%), SMAD4 (32%), FBXW7 (28%), STK11 (28%), MET (15%), SMARCB1 (15%) and ALK (12%) were less frequently altered. In addition, low frequency mutations (<10%) were identified in ABL1 (8%), KIT (5%), NOTCH1 (5%), SMO (5%), ERBB2 (2%) and IDH1 (2%).

We then sought to compare the prevalence of mutations in PBMC samples from patients with advanced HCC stages to those in NTAT samples from patients in early stage disease. The results showed that mutations in FGFR3 and PDGFRA were present in 100% of both PBMC and NTAT samples, while RET was found in 100% of PBMC and 99% of NTAT samples.

Significant differences in mutation frequencies were observed between PBMC and NTAT samples. APC mutations were found in 81% of PBMC samples compared to 100% of NTAT samples, while TP53 mutations were found in 69% of PBMC samples compared to 100% of NTAT samples. Similarly, EGFR mutations were identified in 37% of PBMC compared to 100% in NTAT.

In contrast, several genes showed a higher mutation frequency in PBMC compared to NTAT. These included KDR (100% in PBMC vs. 28% in NTAT), NPM1 (81% in PBMC vs. 50% in NTAT), CSFR1 (100% in PBMC vs. 7% in NTAT), ERBB4 (81% in PBMC vs. 21% in NTAT), HRAS (100% in PBMC vs. 50% in NTAT) and FLT3 (62% in PBMC vs. 31% in NTAT). Mutations in PIK3CA, SMAD4, FBXW7, STK11, MET, SMARCB1, and ALK were predominantly detected in PBMC.

### 3.3 HCC germline variants

The present cohort showed a broad range of germline variants identified in both coding regions (exons) and non-coding regions (intron or 3′UTR) of genes. These mutations included synonymous, non-synonymous, and frameshift variants. Additionally, in some cases, multiple variants were detected within the same gene in the same patient as shown in Table 2.

**TABLE 2:** List of actionable germline mutations detected in HCC patients.

Gene	Position (hg19)	Variant	REF	ALT	Genotype Hom/Het	Variant type	rs ID	Pathogenicity (ClinVar)	Number of cases (%)
IDH1	Chr2: 209113207	NM_005896.3 c.293_300del p.(Thr98LysfsTer43)	TCGTATG	-	0/1	Frameshift indels Exon: 4/10	-	Likely path	1/40 (2%)
ERBB4	Chr2: 212576875	NM_005235.2 c.1024T>C (p.Leu342=)	A	G	1/0	Synonymous Exon: 9/28	rs148466450	Likely benign	1/40 (2%)
ERBB4	Chr2: 212578380	NM_005235.2 c.884-7del	A	-	1/13	Splice region Intron	rs67894136	Benign	14/40 (35%)
ERBB4	Chr2: 212812097	NM_005235.2 c.421 + 58A>G	T	C	0/11	Intron	rs839541	Benign	11/40 (28%)
ALK	Chr2: 29432625	NM_004304.4 c.3836 + 27G>T	C	A	0/5	Intron	rs3738868	Benign	5/40 (12%)
PIK3CA	Chr3: 178917005	NM_006218.3 c.352 + 40A>G	A	G	2/11	Intron	rs3729674	Benign	13/40 (32%)
PIK3CA	Chr3: 178927410	NM_006218.3 c.1173A>G p.(Ile391Met)	A	G	0/6	Missense Exon: 7/21	rs2230461	Benign	6/40 (15%)
FGFR3	Chr4: 1807894	NM_001163213.1 c.1959G>A p.(Thr653=)	G	A	40/0	Synonymous exon: 14/18	rs7688609	Likely benign	40/40 (100%)
PDGFRA	Chr4: 55152040	NM_006206.5 c.2472C>T p.(Val824=)	C	T	0/7	Synonymous Exon: 18/23	rs2228230	Benign	7/40 (17%)
PDGFRA	Chr4: 55141055	NM_006206.5 c.1701A>G p.(Pro567=)	A	G	28/12	Synonymous Exon: 12/23	rs1873778	Likely benign	40/40 (100%)
KIT	Chr4: 55593464	NM_000222.2 c.1621A>C p.(Met541Leu)	A	C	0/2	Missense Exon: 10/21	rs3822214	Likely benign	2/40 (5%)
KDR	Chr4: 55962545	NM_002253.2 c.2615-37dup	-	G	0/10	Intron	rs3214870	Benign	10/40 (25%)
KDR	Chr4: 55980239	NM_002253.2 c.798 + 54G>A	C	T	12/16	Intron	rs7692791	Benign	28/40 (70%)
FBXW7	Chr4: 153247278	NM_018315.4 c.1284A>G p.(Gln428=)	T	C	0/11	Synonymous Exon: 9/11	rs147462419	Likely benign	11/40 (28%)
APC	Chr5: 112175770	NM_000038.5 c.4479G>A p.(Thr1493=)	G	A	21/16	Synonymous Exon: 16/16	rs41115	Benign	37/40 (92%)
CSF1R	Chr5: 149433596	NM_005211.3 c.*35_*36delinsTC	TG	GA	21/4	3-Prime UTR Exon: 22/22	rs2066933	Benign	25/40 (62%)
NPM1	Chr5: 170837514	NM_002520.6 c.847-5del	T	-	0/28	Intron	rs34323200	Benign	28/40 (70%)
EGFR	Chr7: 55249063	NM_005228.4 c.2361G>A p.(Gln787=)	G	A	18/12	Synonymous Exon: 20/28	rs1050171	Benign	30/40 (75%)
MET	Chr7: 116339672	NM_001127500.2 c.534C>T p.(Ser178=)	C	T	0/2	Synonymous Exon: 2/21	rs35775721	Benign	2/40 (5%)
MET	Chr7: 116340269	NM_001127500.2 c.1131C>T p.(Ile377=)	C	T	0/5	Synonymous Exon: 2/21	rs28444388	Benign	5/40 (12%)
MET	Chr7: 116340223	NM_001127500.2 c.1085T>C p.(Met362Thr)	C	T	0/2	Missense Exon: 2/21	rs77523018	Benign	2/40 (5%)
SMO	Chr7:12885364	NM_005631.4 c.1627_1632del p.(Leu543_Ile544del)	CTCATC	-	0/2	Inframe deletion Exon: 9/12	-	VUS	2/40 (5%)
ABL1	Chr9: 133747531	NM_007313.2 c.897_904del p.(Glu300LeufsTer14)	GTGGAAGA	-	0/3	Frameshift indels Exon: 5/11	-	Likely path	3/40 (8%)
NOTCH1	Chr9: 139390877	NM_017617.4 c.7314G>A p.(Pro2438=)	C	T	0/2	Synonymous Exon: 34/34	rs370442918	Likely benign	2/40 (5%)
RET	Chr10: 43613843	NM_020975.4 c.2307G>T p.(Leu769=)	G	T	35/4	Synonymous Exon: 13/20	rs1800861	Benign	39/40 (98%)
HRAS	Chr11: 534242	NM_005343.3 c.81T>C p.(His27=)	A	G	3/20	Synonymous Exon: 2/6	rs12628	Benign	23/40 (57%)
FLT3	Chr13: 28602292	NM_004119.2 c.2053 + 23A>G	T	C	0/5	Intron	rs75580865	Benign	5/40 (12%)
FLT3	Chr13: 28610183	NM_004119.2 c.1310–3T>C	A	G	2/18	Splice region Intron	rs2491231	Benign	20/40 (50%)
TP53	Chr17: 7578210	NM_000546.5 c.639A>G p.(Arg213=)	T	C	0/4	Synonymous Exon: 6/11	rs1800372	Benign	4/40 (10%)
TP53	Chr17: 7579472	NM_000546.5 c.215C>G p.(Pro72Arg)	G	C	17/18	Missense Exon: 4/11	rs1042522	VUS	35/40 (88%)
ERBB2	Chr17: 37881418	NM_004448.3 c.2610G>A p.(Leu870=)	G	A	0/1	Synonymous Exon: 21/27	rs757372006	Likely benign	1/40 (2%)
SMAD4	Chr18: 48603034	NM_005359.5 c.1335A>G p.(Arg445=)	A	G	1/2	Synonymous Exon: 11/12	-	Likely benign	3/40 (7%)
SMAD4	Chr18: 48591923	NM_005359.5 C.1086T>C p.(Phe362=)	T	C	2/11	Synonymous Exon: 9/12	rs1801250	Likely benign	13/40 (32%)
STK11	Chr19 : 1220321	NM_000455.4 c.465–51T>C	T	C	0/11	Intron	rs2075606	VUS	11/40 (28%)
SMARCB1	Chr22: 24176287	NM_003073.4 c.1119-41G>A	G	A	1/5	Intron	rs5030613	Benign	6/40 (15%)

Among the most frequently mutated genes, FGFR3 variant (rs7688609, c.1959G>A, p.Thr653=) was detected in 100% of patients (40/40). This synonymous mutation in exon 14 is classified as likely benign, suggesting it does not alter protein function. Similarly, PDGFRA variant (rs1873778, c.1701A>G, p.Pro567=) was also present in 100% of cases, occurring in exon 12, and classified as likely benign. Additionally, the RET variant (rs1800861, c.2307G>T, p.Leu769=) was identified in 98% of cases (39/40). This synonymous mutation in exon 13, classified as benign.

Furthermore, we have also identified mutations in tumor suppressor genes, including the APC variant (rs41115, c.4479G>A, p.Thr1493=), which was detected in 92% of patients (37/40). This synonymous mutation in exon 16, classified as benign. Moreover, TP53, a key tumor suppressor gene, showed two distinct variants in our cohort. The rs1042522 (c.215C>G, p.Pro72Arg), a missense mutation in exon 4, was detected in 88% of cases (35/40) and classified as variant of uncertain significance (VUS). The second variant, rs1800372 (c.639A>G, p.Arg213=), is a synonymous mutation in exon 6, present in 10% of cases (4/40) and classified as benign (Figure 1b).

Frequent mutations were also detected in genes encoding tyrosine kinase receptors, including the EGFR variant (rs1050171, c.2361G>A, p.Gln787=), which was identified in 75% of patients (30/40). This synonymous mutation in exon 20 is classified as benign. Additionally, KDR variant (rs7692791, an intronic mutation) was found in 70% of cases (28/40), while NPM1 variant (rs34323200, an intronic mutation) exhibited a similar frequency of 70% (28/40). Although these mutations are classified as benign (Figure 1b).

Furthermore, ERBB4, a member of the EGFR family, exhibited two frequently mutated variants. The first variant, rs839541 (c.421 + 58A>G, intronic), was found in 28% of cases (11/40) and classified as benign. The second variant, rs67894136 (c.884-7del, splice region), was present in 35% of cases (14/40) and also classified as benign. In addition, the CSF1R variant (rs2066933, 3′ UTR variant) was mutated in 62% of cases (25/40). This variant can affect mRNA stability and translation efficiency, potentially leading to changes in CSF1R protein expression. Similarly, HRAS variant (rs12628, c.81T>C, p.His27=) was present in 57% of cases (23/40). Although classified as benign, HRAS is a well-established oncogene involved in MAPK signalling, a critical pathway in cell proliferation and survival.

**FIGURE 2:**
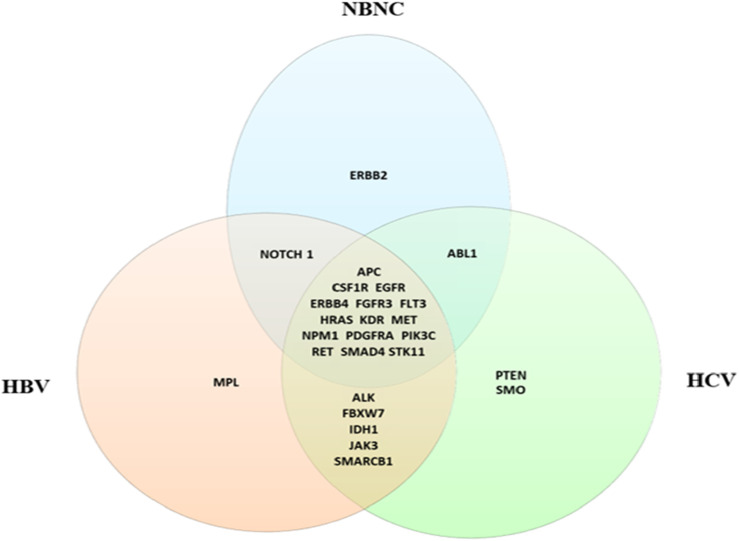
Venn diagram shows the distribution of germline mutations in HCC cases stratified by etiology. Shared mutations across all groups include key oncogenes such as *APC*, *EGFR*, and *PIK3CA*. Etiology-specific mutations include *MPL* (HBV), *PTEN* (HCV), and *ERBB2* (NBNC).

The Venn diagram in Figure 2 illustrates the mutational landscape of HCC across different etiologies in our cohort, highlighting both shared and etiology-specific genetic alterations. Mutations in APC, CSF1R, EGFR, ERBB4, FGFR3, FLT3, HRAS, KDR, MET, NPM1, PDGFRA, PIK3CA, RET, SMAD4 and STK11 were detected in HCC patients across all etiologies. In contrast, ERBB2 mutations were exclusively identified in the NBNC group, whereas PTEN and SMO mutations were specific to the HCV-associated HCC group. Similarly, MPL mutations were uniquely found in the HBV-associated HCC group. Additionally, mutations in ALK, FBXW7, IDH1, JAK3, and SMARCB1 were observed in post-viral HCC cases (HBV and HCV).

### 3.4 Somatic mutations identified in HCC patients

**FIGURE 3:**
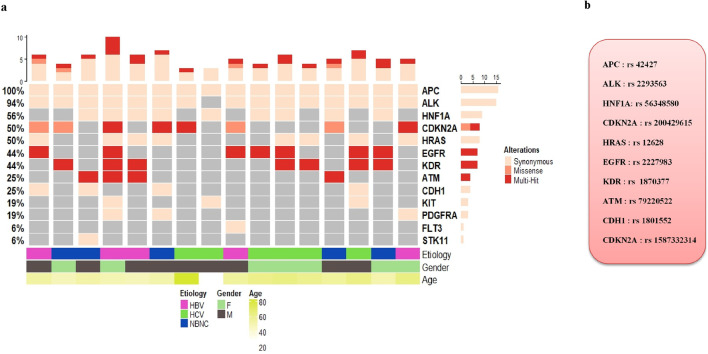
**(a)** Oncoplot displaying hotspot oncogenic somatic mutations that passed stringent filtering criteria, identified in liver tumor tissues by comparison with matched adjacent non-tumor tissues. Each column represents an HCC sample from individual after liver resection, and each row corresponds to a cancer-associated gene. **(b)** List of the most frequently oncogenic hotspot somatic mutations detected in HCC tissues.

The oncoplot in Figure 3a provides an overview of the somatic mutation landscape detected in the tumor tissues of HCC patients across different etiologies. The analysis reveals that APC is the most frequently mutated gene (100%), followed by ALK (94%), then HNF1A (56%). Other genes showing notable mutation frequencies include CDKN2A and HRAS (50%). In contrast, genes such as EGFR and KDR show moderate mutation frequencies (44%), while ATM and CDH1 are mutated in 25% of cases. Less frequently mutated genes include KIT and PDGFRA (19%), as well as FLT3 and STK11 (6%).

To compare the frequencies of somatic mutations detected in our study with those reported in other global populations, we used data from cBioPortal. The comparative mutation frequencies for shared genes were as follows: APC (100% vs. 0.2%), ALK (94% vs. 0.4%), HNF1A (56% vs. 0.2%), CDKN2A (50% vs. 4%), HRAS (50% vs. 0.4%), EGFR (44% vs. 1.3%), KDR (44% vs. 0.2%), ATM (25% vs. 0.5%), CDH1 (25% vs. 0%), KIT (19% vs. 0.1%), PDGFRA (19% vs. 0.2%), FLT3 (6% vs. 0.3%), and STK11 (6% vs. 0.6%). Notably, the frequencies of mutated genes in our cohort were consistently higher than those reported in cBioPortal.

Furthermore, our results showed distinct pathogenicity profiles for the somatic mutations identified in HCC tumor tissues, as shown in Table 3. The somatic SNPs detected in APC (rs42427), ALK (rs2293563), HNF1A (rs56348580), and HRAS (rs12628) were classified as having undetermined pathogenicity, indicating that their functional impact on tumorigenesis remains unclear and requires further investigation. In contrast, the mutations identified in EGFR (rs2227983) and KDR (rs1870377) were classified as benign. However, the mutations detected in CDKN2A (rs200429615 and rs1587332314) and ATM (rs79220522) were classified as deleterious, implying a detrimental effect on protein function and a potential role in HCC pathogenesis (Figure 3b).

**TABLE 3:** List of actionable somatic mutations detected in HCC patients.

Gene	Position (hg19)	Variant	REF	ALT	Genotype Hom/Het	Variant type	rs ID	Pathogenicity (ClinVar)	Number of cases (%)
ALK	Chr2: 29449819	NM_004304.4 c.3036G>A p.(Thr1012=))	C	T	8/7	Synonymous Exon: 18/29	rs2293563	NA	15/16 (94%)
KDR	Chr4: 55972974	NM_002253.2 c.1416A>T p.(Gln472His)	T	A	2/5	Missense Exon: 11/30	rs1870377	Benign	7/16 (44%)
KIT	Chr4: 55599268	NM_000222.2 c.2394C>T p.(Ile798=))	C	T	0/3	Synonymous Exon: 17/21	rs55789615	NA	3/16 (19%)
PDGFRA	Chr4: 55152040	NM_006206.5 c.2472C>T p.(Val824=))	C	T	0/3	Synonymous Exon: 18/23	rs2228230	NA	3/16 (19%)
APC	Chr5: 112176325	NM_000038.5 c.5034G>A p.(Gly1678=))	G	A	10/6	Synonymous Exon: 16/16	rs42427	NA	16/16 (100%)
EGFR	Chr7: 55229255	NM_005228.4 c.1562G>A p.(Arg521Lys)	G	A	2/5	Missense Exon: 13/28	rs2227983	Benign	7/16 (44%)
CDKN2A	Chr9: 21971137	NM_000077.4 c.221A>C p.(Asp74Ala)	T	G	3/5	Missense Exon: 2/3	rs200429615	Deleterious	8/16 (50%)
CDKN2A	Chr9: 21971164	NM_000077.4 c.194T>C p.(Leu65Pro)	A	G	0/4	Missense Exon: 2/3	rs1587332314	Deleterious	4/16 (25%)
ATM	Chr11: 108121428	NM_000051.3 c.1236G>T p.(Trp412Cys)	G	T	0/4	Missense Splice region Exon: 10/63	rs79220522	Deleterious	4/16 (25%)
HRAS	Chr11: 534242	NM_005343.3 c.81T>C p.(His27=))	A	G	4/4	Synonymous Exon: 2/6	rs12628	NA	8/16 (50%)
HNF1A	Chr12: 121432117	NM_000545.6 c.864G>C p.(Gly288=))	G	C	0/9	Synonymous Exon: 4/10	rs56348580	NA	9/16 (56%)
FLT3	Chr13: 28608459	NM_004119.2 c.1683A>G p.(Leu561=))	T	C	0/1	Synonymous Exon: 13/24	rs34374211	NA	1/16 (6%)
CDH1	Chr16: 68835787	NM_004360.4 c.378G>C p.(Pro126=))	G	C	0/4	Synonymous Exon: 13/16	rs1801552	NA	4/16 (25%)
STK11	Chr19: 1221293	NM_000455.4 c.816C>T p.(Tyr272=))	C	T	0/1	Synonymous Exon: 6/10	rs9282859	NA	1/16 (6%)

### 3.5 PPI networks analysis of somatic mutated genes

**FIGURE 4:**
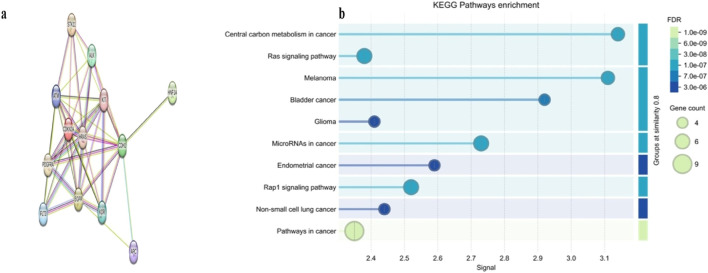
Protein–protein interaction (PPI) networks and KEGG pathway enrichment of somatic mutated genes in our HCC cohort. **(a)** PPI network somatically mutated genes, highlighting CDKN2A as a central hub within the interaction landscape. **(b)** KEGG pathway enrichment analysis of somatic mutated genes, showing significant enrichment in central carbon metabolism and other cancer-related pathways.

To investigate the functional interplay among genes altered in HCC, we constructed PPI networks for somatic mutations using the STRING database. The PPI network generated from somatic mutations included 13 nodes and 46 edges, exceeding the expected number of 13 edges (PPI enrichment p-value <0.001). This network displayed a high average node degree of 7.08 and a clustering coefficient of 0.855, with CDKN2A as the central query protein (Figure 4a). A major functional cluster was identified and enriched in central carbon metabolism in cancer, positive regulation of phospholipase C activity, and transmembrane receptor protein tyrosine kinase activity (Figure 4b). These findings suggest that somatic alterations in our HCC cohort converge on interconnected oncogenic and metabolic pathways that may contribute to tumor development and progression.

## 4 Discussion

HCC is a highly complex and heterogeneous malignancy, both clinically and molecularly. Its pathogenesis is influenced by a various etiological factors, including chronic viral infections, metabolic disorders, and exposure to environmental carcinogens (Llovet et al., 2021). In addition to these factors, host genetic components play a crucial role in the predisposition and initiation to liver cancer. Genetic alterations encompass both somatic mutations, acquired during tumor development, and inherited germline alterations that may increase susceptibility to carcinogenesis. This molecular pathogenesis heterogeneity of HCC is reflected in the diverse landscape of genetic aberrations affecting key oncogenes, tumor suppressor genes, and regulatory signalling pathways (Schulze et al., 2015; Llovet et al., 2023).

High-throughput genomic studies have revealed recurrent mutations and dysregulation of critical molecular pathways, notably involving TP53, CTNNB1, TERT, and signalling cascades as the Wnt/β-catenin, PI3K/AKT/mTOR, and MAPK. Despite these advances, most of these mutations remain clinically non-actionable, and their characterization has yet to translate into improved management strategies for HCC (Llovet et al., 2021). This limitation highlights the pressing need for continued research to uncover novel driver mutations, particularly within oncogenes and tumor suppressor genes, using advanced sequencing technologies and refined analytical approaches. Such efforts are particularly important in underrepresented populations, where the molecular landscape of HCC remains insufficiently characterized.

In this context, our study aims to identify both germline and somatic mutations associated with HCC development in the Tunisian population, which serves as a representative cohort for the broader North African region. To achieve this, we employed a targeted NGS approach utilizing an optimized gene panel that screens 2,800 hotspot mutations across 50 well-established cancer predisposition genes.

Through sequencing analysis of non-tumoral liver tissues and PBMC from patients with HCC, we identified a spectrum of germline variants potentially associated with genetic susceptibility to HCC. Among these, we observed high-frequency variants across 25 cancer-related genes, with complete penetrance notably found for FGFR3 (rs7688609, COSM4533173) and PDGFRA (rs1873778, COSM7410554). These two polymorphisms, although synonymous, are located in critical exonic regions of their respective oncogenes exon 14 of FGFR3 (c.1959G>A, p.Thr653=) and exon 12 of PDGFRA (c.2472C>T, p.(Val824=)). Importantly, both FGFR3 and PDGFRA are known to play pivotal roles in hepatocarcinogenesis. Overexpression of these genes has been previously associated with enhanced proliferation, angiogenesis, and progression of HCC (Wei et al., 2014; Paur et al., 2015). While these mutations have not been previously identified in HCC, they have recently been described in several other types of cancer, including osteosarcoma (Chantre-Justino et al., 2025), and colorectal cancer (Estevez-Garcia et al., 2012). Furthermore, we observed the RET variant (rs1800861, COSM4418405) in 98% of patients, reaching 100% prevalence in advanced-stage of HCC, mirroring its known associations with adamantinomatous craniopharyngioma (Jastania et al., 2020), thyroid cancer (Minna et al., 2022) and chronic myeloid leukemia (Sklarz et al., 2018). The APC variant (rs41115, COSM3760869) exhibited stage-dependent prevalence (92% overall; 87% in advanced vs. 100% in early-stage HCC) and has been reported in colorectal cancer (Manirakiza et al., 2023). Intriguingly, EGFR (rs1050171, COSM1451600) was present in 75% of the cohort overall, it showed complete penetrance (100%) in early-stage HCC, consistent with reports in glioblastoma (Loriguet et al., 2018) and esophageal cancer (Showeil et al., 2016). While these variants have been identified in several cancers, these studies analysed them only in tumor tissues, leaving their classification as somatic or germline mutations unresolved. However, in this study, paired sequencing of tumor and adjacent healthy tissues confirmed their germline origin within our cohort. Although these five high-prevalence synonymous variants have not been previously reported in HCC, they were consistently detected at high prevalence in both early- and advanced-stage HCC in our cohort. This finding suggests that, while these germline variants do not appear to be oncogenic drivers, their high prevalence within our cohort may reflect underlying genetic patterns that warrant further investigation regarding potential predisposition to HCC development.

In addition, our analysis identified a germline missense variant in the tumor suppressor gene TP53 (rs1042522, COSM250061; Pro72Arg), which was present in 100% of early-stage HCC patients and 80% of advanced-stage cases. The TP53 gene plays a central role in maintaining genomic stability, regulating cell cycle arrest at the G1 phase, promoting apoptosis in response to DNA damage, and preventing malignant transformation. Codon 72 of TP53 (rs1042522) is one of the most polymorphic sites within the gene and results in a functional substitution of proline (Pro) by arginine (Arg). This variant has been reported to modulate p53 activity, where the Arg72 variant exhibits a higher apoptotic potential, whereas the Pro72 variant is more efficient at inducing cell cycle arrest (Dumont et al., 2003). The functional impact of this polymorphism has been linked to increased susceptibility to several cancers, including HCC (Hu et al., 2014). Furthermore, the Arg72 allele has been associated with a higher frequency of somatic TP53 mutations in tumors and has been shown to induce gene expression programs related to enhanced cell proliferation and activation of pro-oncogenic signalling pathways (Soussi and Wiman, 2007; De Souza et al., 2021). These findings suggest a potential role for this variant in promoting genomic instability and contributing to tumor progression (Soussi and Wiman, 2007). In the same context, Rebbani et al. proposed that the Arg72 polymorphism may act as a primary driver of epigenetic alterations in HCC (Rebbani et al., 2015). Moreover, recent case–control studies conducted in the Tunisian population have shown that this variant is associated with several types of cancer, including chronic lymphocytic leukemia and cervical cancer (Ounalli et al., 2023; Ben Jemia et al., 2025). Based on our findings and previous reports, we propose that the presence of the TP53 variant, in combination with other germline mutations identified in this study, may contribute to a cascade of molecular events that collectively promote the initiation and progression of HCC. These results underscore the potential role of inherited genetic factors in shaping the molecular landscape of HCC. They also highlight the critical need to further investigate germline susceptibility, particularly in North African populations.

On the other hand, we report the somatic mutational landscape of HCC in our cohort, identified exclusively in the tumor tissues, with APC (100%), ALK (94%), HNF1A (56%), CDKN2A and HRAS (50%) emerging as the most frequently mutated genes.

Mutations in the APC gene, a key negative regulator of the Wnt/β-catenin signalling pathway, are well-known drivers of tumorigenesis and have been increasingly reported in HCC (Xu et al., 2022; Bugter et al., 2021). The relatively high frequency of APC mutations observed in our cohort appears to contradict previous reports, which indicate that APC mutations occur in only 3% of HCC cases, with CTNNB1 and AXIN1 being the most frequently altered components of the Wnt/β-catenin pathway (Xu et al., 2022). However, the absence of detectable mutations in CTNNB1 in our cohort may be explained by both technical and biological factors. From a technical perspective, the limited genomic coverage of our targeted sequencing panel may have restricted the detection of CTNNB1 variants, which are more reliably captured through whole-exome sequencing (WES). Biologically, CTNNB1 mutations are often associated with alcohol-related HCC (Llovet et al., 2018), a profile not represented in our study population. In addition, it has been reported that driver mutations in oncogenic genes such as KRAS, TP53, SMAD4 and PIK3CA, can cooperate with APC mutations to promote the stepwise progression from adenoma to carcinoma in colorectal cancer (Bugter et al., 2021). Based on our findings, we hypothesize that APC mutations may similarly cooperate with driver mutations in oncogenic genes such as ALK, HNF1A, CDKN2A, HRAS, and EGFR to initiate an alternative oncogenic route leading to HCC in our study population.

Comparison of our somatic mutation findings with genomic data reported in other populations reveals both similarities and notable differences. In our cohort, the CDKN2A gene exhibited a high frequency (50%) of deleterious somatic missense mutations (rs200429615, p.Asp74Ala). This gene has also been reported as mutated in a WES study involving European HCC patients, although with a different pathogenic nonsense mutation (rs121913384, p.Glu88*) (Guichard et al., 2012). Interestingly, the rs200429615 variant detected in our cohort has been described as a recurrent hotspot mutation in an Indian breast cancer cohort, where it was classified as a driver mutation (Nirgude et al., 2023).

However, the somatic missense mutation detected in the EGFR gene (rs2227983, p.Arg521Lys), present in 44% of our cohort, has not been reported in large scale genomic studies of HCC. Notably, this polymorphism has been previously associated with HBV-related HCC (Han et al., 2016). In addition, the KDR gene (encoding VEGFR2 receptor), has been reported to modulate the therapeutic response to regorafenib (Teufel et al., 2019). The missense variant (rs1870377, p.Gln472His), detected in 44% of patients in our cohort, has been associated with an increased risk of tumor recurrence and with treatment outcomes in advanced HCC (Teufel et al., 2019; Zheng et al., 2014). Recent evidence further indicates that VEGFR2 enhances CD8^+^ T-cell recruitment through activation of the PI3K/AKT/HIF-α pathway following anlotinib treatment in HCC (Song et al., 2024). Together, these findings underscore the pivotal role of *KDR* in HCC biology and highlight the relevance of identifying functionally impactful variants in this gene, which may contribute to disease progression and shape therapeutic responses. Finally, somatic mutations in the ATM gene have been reported in a whole-genome sequencing (WGS) study of Asian HCC patients; however, the variant identified in our cohort (rs79220522, p.Trp412Cys) was not identified in that study (Fujimoto et al., 2012). In silico predictions suggest that this mutation may impact splicing (Grodecká et al., 2014). These results reveal, for the first time, a distinct mutational profile associated with HCC in a North African population. The recurrent somatic alterations identified, both shared with other global cohorts and unique to Tunisian patients, suggest the existence of population-specific molecular mechanisms driving hepatocarcinogenesis, which may be associated with ethnic differences or its significant intertumoral and intratumoral heterogeneity. Altogether, these findings expand the current genomic landscape of HCC and highlight the need for further studies to validate these variants and explore their implications in HCC development.

Furthermore, analysis of somatic mutated genes using STRING and KEGG pathway enrichment suggested that central carbon metabolism in cancer is affected in our HCC cohort. This is consistent with recent studies demonstrating that dysregulation of metabolic pathways, including glycolysis, gluconeogenesis, and lipid metabolism, represents a hallmark of hepatocarcinogenesis (Xia et al., 2022; Park and Hall, 2025). Moreover, studies have suggested that metabolic reprogramming in HCC is remarkably heterogeneous due to diverse driver gene mutations and oncogenic signals in liver cancer subsets (Yang et al., 2023), which highlights the importance of comprehensive detection of such mutations across different populations is to better unravel the molecular complexity of this disease. This knowledge could pave the way for precision medicine approaches, where therapeutic strategies are tailored to the specific genetic and metabolic profiles of individual patients.

In summary, this study is the first to demonstrate the interplay between germline and somatic variants in shaping the molecular heterogeneity of HCC in the Tunisian population. These findings underscore the importance of investigating underrepresented populations to uncover distinct oncogenic mechanisms. While the relatively small sample size remains a limitation, the genetic profiles identified here strongly support the extension of sequencing efforts to larger cohorts and additional genomic regions, which will be crucial for uncovering HCC-associated mutations specific to Tunisian patients.

## Data Availability

The datasets presented in this article are not readily available due to ethical and legal restrictions under Tunisian law concerning human genomic data and participant privacy. Requests to access the datasets should be directed to the corresponding author.
